# Changing landscapes for plastic surgery: the effect of the Major Trauma Network on emergency operative workload

**DOI:** 10.1186/1757-7241-23-S2-O1

**Published:** 2015-09-11

**Authors:** Susan A Hendrickson, Denise Osei-Kuffour, Kazi Rahman, Jonathan Simmons, Shehan Hettiaratchy

**Affiliations:** 1 Major Trauma Centre, St. Mary's Hospital, Imperial College Healthcare NHS Trust, London, UK

## Background

The advent of major trauma centres (MTCs) in the UK in 2010 has led to a concentration of complex, polytrauma cases in these centres. The role plastic surgeons play in trauma has increased and evolved over time [1], and currently plastic surgeons input into a wide variety of trauma [2]. Our study aimed to analyse the effect of MTC status on plastic surgery activity at our centre.

## Method

All trauma patients admitted to a London MTC in 2013 who underwent an operation were identified using Trauma Audit & Research Network data. Operative procedure(s) and operating specialty were recorded. This was compared to local historical data from pre-MTC go-live (2008–2010).

## Results

Of the 2606 trauma calls in 2013, 416 patients required surgical intervention. 29.3% of these patients (n = 122) were operated on by plastics (either as sole operating team or part of multi-specialty team). 76.2% (n = 93) involved lower limb trauma and 30.3% (n = 37) upper limb trauma. Emergency general extremity referrals increased from an average of 65/year to 484/year in the period 2011 to 2013, whilst plastics operative workload increased from an average of 53 cases/year to 407/year in the same period. This represents a more than sevenfold increase in the plastic surgery operative workload at our centre.

## Conclusion

There has been a dramatic increase in emergency plastic surgery activity following designation of major trauma centre status at our centre. Understanding the epidemiology of plastic surgery is vital to improve service design, postgraduate training in the specialty, and workforce provision [1].
